# Early Intervention and Children’s Academic Outcomes—Why Part C Matters

**DOI:** 10.1001/jamanetworkopen.2025.55776

**Published:** 2026-02-02

**Authors:** Anisha P. Srinivasan, Yuuko Mabrey Johnson, Aubyn C. Stahmer

**Affiliations:** Department of Pediatrics, MIND Institute, University of California, Davis, Sacramento; Betty Irene Moore School of Nursing, University of California, Davis, Sacramento; Department of Psychiatry & Behavioral Sciences, MIND Institute, University of California, Davis, Sacramento

Federally funded early intervention (EI) programs through Part C of the Individuals with Disabilities Education Act (IDEA) aim to “enhance the development of infants and toddlers with disabilities and to minimize their potential for developmental delay.”^1^ The study by Stingone et al^2^ provides compelling and timely evidence that EI programming is associated with population-level improvements in later educational outcomes.

The investigators evaluated third-grade academic outcomes among children born in New York City between 1994 and 1998, comparing those who received services through the EI program with those who did not. The study population included 214 370 children with available third-grade academic records through 2007. Notably, the study linked EI program data with Department of Education records, systems that are typically administratively separate. The primary exposure was receipt of any EI services before 3 years of age, and primary outcomes were third-grade standardized test scores in mathematics and English Language Arts (ELA). Third grade is a key assessment point as children transition from “learning to read” to “reading to learn,” with not meeting literacy benchmarks at this stage being associated with increased risk of not completing high school.^3^ To reduce confounding and enhance between-group exchangeability, investigators used propensity score matching to compare outcomes between EI recipients and nonrecipients. This quasi-experimental approach was novel and necessary given the ethical and legal constraints precluding randomization of policy-mandated EI services.

Results showed that EI recipients had greater likelihood of meeting third-grade test standards in ELA and math relative to nonrecipients. The magnitude of association was greatest among the subgroup that was determined to need special education services after 3 years of age (28% and 17% increased likelihood for ELA and math, respectively). The finding that children who received both EI and special education demonstrated better academic outcomes than matched peers who received special education alone suggests that EI may provide foundational benefits that could enhance the effectiveness of subsequent educational services.

The study’s subgroup analyses also showed significant association between EI and academic outcomes among children of mothers without high school or college completion (8% to 13% increased likelihood), Latina mothers born outside the US (15% to 18%), and mothers with public insurance (10% to 13%). This is noteworthy given the well-documented racial, ethnic, linguistic, and socioeconomic disparities in EI service uptake.^4,5^ These findings highlight the need to address systemic barriers to EI participation so that children who may benefit most from EI can equitably access these services. A helpful next step might beto better understand how additional supports (eg, language interpretation, service navigation, transportation) can improve EI access for families born outside the US or those with limited financial resources.

The study by Stingone et al^2^ examines outcomes for children participating in EI when the services first became publicly available. Since that time, tools available for teaching young children with developmental disabilities have improved.^6^ The 1990s represented a period of strong advocacy for policies to support learning for all children—which is how EI services began to be mandated. Soon after, educators began calling for an understanding of best practices for use in these mandated programs. Since the early 2000s, evidence-based practices, which show efficacy in research studies, have been available for use in education programs across the age range. While a research-to-practice gap still exists, our ability to effectively identify and support children with developmental disabilities has increased over the past several decades, suggesting that the academic benefits associated with EI may be even greater today.

Limitations include the lack of data on neurodevelopmental diagnoses (eg, intellectual disability) and inability to assess outcomes for children exempted from third-grade testing. In New York state, up to 1% of students with severe disabilities, defined as those with limited cognitive abilities combined with behavioral and/or physical limitations and requiring specialized services, are eligible to take an alternative assessment.^7^ Therefore, children with the most severe disabilities may not participate in third-grade testing, which means data from the current study may be most relevant for students with mild to moderate disabilities. However, the study has notable strengths, including the use of population-level linked administrative data and quasi-experimental design, which facilitates stronger causal inference than conventional observational approaches.

The study advances the science on EI, which has primarily documented improvements in developmental functioning, by being one of the first to examine longer-term academic outcomes of policy-mandated programming.^8^ The authors’ contribution toward substantiating the value of Part C IDEA programs is timely given current federal actions toward relocation of authority and oversight of special education to the Department of Health and Human Services. While various early intervention models have demonstrated efficacy, evidence that federally funded EI services—accessible nationwide regardless of geography—are associated with improvements in school-age academic performance suggest that public programs may confer lasting benefits. These results underscore the importance of publicly funded EI in supporting positive educational outcomes.

## References

[1] Library of Congress. The Individuals with Disabilities Education Act (IDEA), Part C: Early Intervention for Infants and Toddlers with Disabilities. February 26, 2024. Accessed November 26, 2025. https://www.congress.gov/crsproduct/R43631

[2] Stingone J, McVeigh K, Lednyak L. Early intervention developmental programming and childhood academic outcomes. JAMA Netw Open. 2026;9(2):e2555890. doi:10.1001/jamanetworkopen.2025.55890 41661596 PMC12887746

[3] Annie E.Casey Foundation. EarlyWarning! Why Reading by the End of Third Grade Matters. 2010. Accessed November 26, 2025. https://assets.aecf.org/m/resourcedoc/AECF-Early_Warning_Full_Report-2010.pdf

[4] Shenouda J, Barrett E, Davidow AL, Disparities in early intervention program participation by children with autism spectrum disorder in a US metropolitan area, 2006 to 2016. JAMA Pediatr. 2022;176(9):906–914. doi:10.1001/jamapediatrics.2022.2366 35849409 PMC9295023

[5] Cycyk LM, De Anda S, Ramsey KL, Sheppard BS, Zuckerman KE.Moving through the pipeline: ethnic and linguistic disparities in special education from birth through age five. Educ Res. 2022;51(7):451–464. doi:10.3102/0013189X221120262 37032722 PMC10078922

[6] Collins LW, Cook SEC, Cook BG. Evidence-based practices in special education: a brief evolution and noteworthy practices. Teach Except Child. Published online October 15, 2025. doi:10.1177/00400599251382310

[7] New York State Education Department. Eligibility criteria for participation in the New York State Alternate Assessment (NYSAA). March 2025. Accessed December 3, 2025. https://www.nysed.gov/sites/default/files/programs/special-education/nysaa-policy-brief.pdf

[8] Elbaum B, Celimli-Aksoy S. Developmental outcomes of children served in a Part C early intervention program. Infants Young Child. 2022;35(1):3–19. doi:10.1097/IYC.0000000000000205

